# A New Surgical Method of Suprapubic and Extraperitoneal Approach with Uterine Preservation for Pelvic Organ Prolapse: Kurt Extraperitoneal Ligamentopexy

**DOI:** 10.1155/2013/748232

**Published:** 2013-12-17

**Authors:** Sefa Kurt, Mehmet Tunc Canda, Abdullah Tasyurt

**Affiliations:** ^1^Tepecik Teaching and Research Hospital, Gynecology and Obstetrics Department, Gaziler Caddesi No. 468, Yenişehir, 35330 Izmir, Turkey; ^2^Kent Hospital, Gynecology and Obstetrics Department, 8229/1 Sokak No. 56, Cigli, 35580 Izmir, Turkey

## Abstract

*Objective*. To introduce an alternative surgical approach for the optimal treatment of pelvic organ prolapse (POP). *Methods*. Twenty symptomatic women with grades 2–4 POP diagnosis who opted to choose this alternative surgery were retrospectively analyzed. *Results*. A total of 22 cases were included. The mean age of the patients was 50.6 ± 13.2 years (29–72 years) with a mean gravid and parity of 5.5 ± 2.66 and 3.4 ± 2.06, respectively. The mean body-mass index of the patients was 24.25 ± 2.43. Nine (45%) patients were premenopausal and 11 (55%) patients were postmenopausal. Uterine descensus was present in all patients, and additionally cystorectocele in 9 patients (45%), cystocele in 6 patients (30%), rectocele in 4 patients (20%), and elangatio colli in 6 patients (30%) were diagnosed. In addition to the alternative surgery, Manchester procedure and anteroposterior vaginal wall repair or Burch procedure was performed where necessary. Mean follow-up time was 48.95 ± 42.8 months (6–171 months). No recurrence of POP occurred. *Conclusions*. Suprapubic, extraperitoneal, and minimally invasive ligamentopexy of the round ligament to the anterior rectus fascia offers an alternative to conventional POP surgery with favorable outcomes without any recurrence.

## 1. Introduction

Pelvic organ prolapse (POP) is the descent of adjacent organs to the vaginal vault through the vagina due to damage to the supportive and suspensory elements of the pelvic floor and endopelvic fascia. Genetic, environmental, and multifactorial determinants play a role in the etiology. The terms cystocele, cystouretherocele, uterine prolapse, rectocele, and enterocele are used to define the place of exit and the anatomic defect [1, 2].

The prevalence varies depending on the geographical regions and advancing age ranging between 11 and 64.8% [3, 4]. POP prevalence increases with prolongation of life in developed countries and improvement of health care services in underdeveloped countries. It is not a mortal disease, but its morbidity is common. POP is the 3rd common cause of hysterectomy for benign conditions with almost one billion dollars treatment costs a year in the United States (US) [5]. Notwithstanding, it generates a remarkable irony that hysterectomy is both the cause and the result of POP. While the hysterectomies are being done for POP increase, evidence related to posthysterectomy POP development is also on the rise [6–8]. Moreover, some women wish to preserve their uterus and look for a surgical opportunity instead of a hysterectomy operation for POP.

Herein, we report a new surgical approach for women suffering from POP but wish to preserve their uterus. This new surgical technique of extraperitoneal ligamentopexy for POP is accompanied with standardized pelvic repair surgery. Our aim in this study is to present our experience with a different surgical approach of uterus preservation for POP surgery with patient selection criteria, definition of the surgical method, and longtime follow-up outcomes.

## 2. Materials and Methods

This retrospective study was performed in Tepecik Teaching Hospital from cases operated in timeline between 1998 and 2012. Women referred to the outpatient gynecology clinic due to symptomatic POP were informed about possible operative alternatives as well as the new procedure Kurt extraperitoneal ligamentopexy (KEPL). Women who choose the KEPL procedure were given their informed consent. A total of 22 patient records that were treated for POP with the KEPL procedure were reached. Two patients were excluded from the study because one patient was lost to followup and the other one was newly operated. Patient selection criteria, applied surgical procedure, postoperative complications, and short and long time outcomes constituted the principals of our study.

POP grading was performed on the lithotomy position with full bladder using Baden-Walker halfway system with one investigator (S.K.) in every case. If exists presence and type of urinary incontinence were also stated. The inclusion criteria for the study were determined as women with symptomatic POP diagnosis with grades 2–4 uterine descensus accompanied by pelvic floor disorders (cystocele and/or rectocele, enterocele), no abnormal finding instead of POP on gynecological and ultrasound examination (normal smear, benign endometrial samplings, sonographically normal uterus, and ovaries with normal CA 125 levels), women not desiring pregnancy or completed their fertility, and choosing the new procedure and its possible complications to the conventional methods. Exclusion criteria consisted of any previous or present gynecological malignity, uterine leiomyomas, present endometriosis or previous endometriosis surgery, and any previous incontinence or POP surgery.

**Figure 1:**
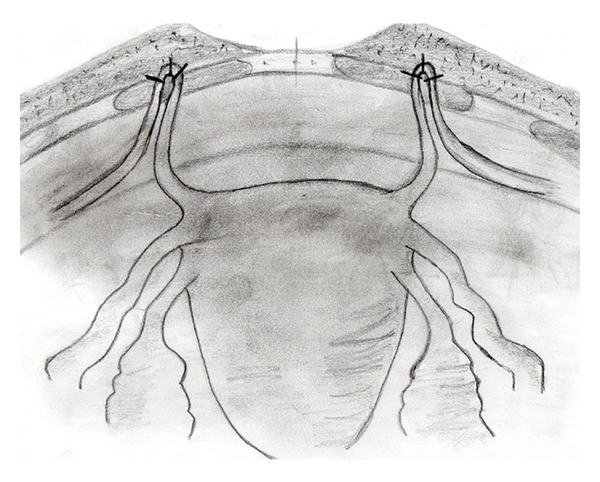
Schematic drawing showing the fixation of the round ligament to the fascia.

**Figure 2:**
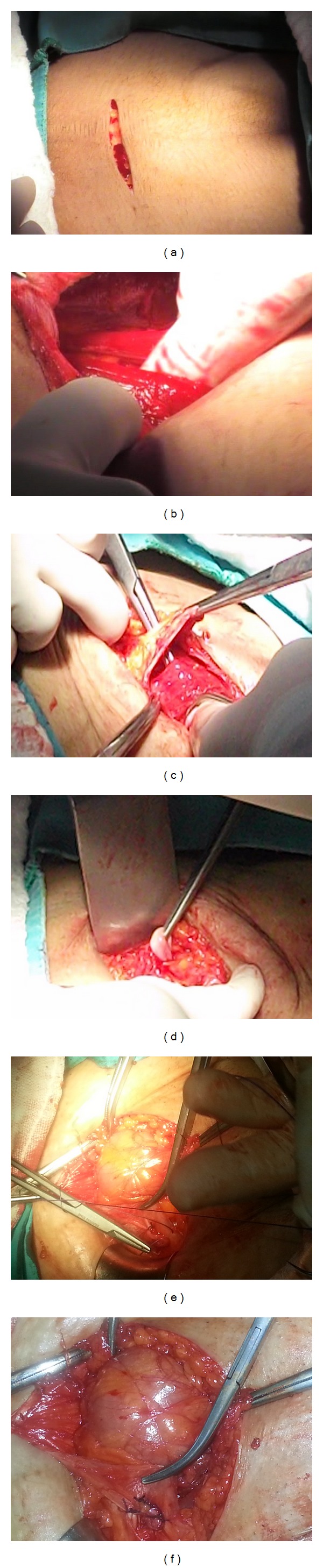
Operation images of the Kurt extraperitoneal ligamentopexy. (a) Suprapubic mini Cherney incision, ((b), (c)) extraperitoneal retzius space dissection, (d) identification of a free ans of round ligament at the entrance of inguinal canal, (e) fixation of the round ligament to the anterior rectus fascia, and (f) fixed round ligament to the fascia from the fenestrated rectus muscle, and note the intact peritoneum with intestines.

The operation is performed in the lithotomy position under general or epidural anesthesia according to patient needs. Povidone-iodine solution was used for skin and vaginal cleansing. Before the KEPL procedure, patients with elangatio colli were treated with Manchester procedure, patients with cystocele were treated with anterior vaginal wall repair, and patients with rectocele or enterocele were treated with posterior vaginal wall repair. While levator plication is avoided in young and sexually active women, perineoplasty and levator plication are performed in older women. After the vaginal procedures, we passed to abdominal approach for the KEPL operation. The patient's abdomen was again scrubbed with povidone-iodine solution, while the patient was still in lithotomy and dorsal supine position. Also, the surgical team cleaned up and dressed again. The skin is opened with a suprapubic mini Cherney, incision using a scalpel and extraperitoneal retzius space was entered with sharp and blunt dissection. Two Langenbeck retractors were used to explore the retzius space. Blunt dissection was performed cranially and laterally with mounted tampon and collet to identify the round ligament at the entrance of inguinal canal. The peritoneum was stroked medially avoiding peritoneal opening to unbind the round ligament and to create a 3-4 cm free ans. While the uterus was repositioned upwards through the vagina by an assistant, the surgeon palpated the uterine fundus over the peritoneal sac to locate the new uterine position and determine the rectus anterior fascia region where the round ligament to be fixed. Rectus muscle was fenestrated so that the round ligament was shouldered over the fascia sheet. This procedure was formed due to the grade of the uterine descensus and the new location of uterus was determined according to where the free round ligament ans to be located to the lower or upper fascial pole of the incision, laterally or medially, and how wide a surface area would be fixed. At least 1.5 cm of free ans of round ligament with at least 3 nonabsorbable monofilament suture materials was fixed to the anterior rectus fascia. A schematic drawing of the technique is given in Figure 1. Operation images of the technique are given in order in Figure 2. Afterwards, the bladder was filled with saline and the preoperative and intraoperative POP grading was compared. Optimal surgery was defined as uterine descensus ≤ grade 1. After the control of the new location of the uterus and bleeding, abdomen was closed appropriate for the anatomy and operation was ended. After the operation in near-term follow-up patients were evaluated for incisional infections, pelvic pain, and analgesia need and in long-term followup for POP recurrence. In the follow-up visits patients were examined in lithotomy position both for the healing of lower genital tract deficit and a possible recurrence of POP with bimanual digital exam, while they were asked to perform valsalva maneuver. Additionally, ultrasonography was performed for the visualization of the location of the uterus.

## 3. Results

The mean age of patients in the study was 50.6 ± 13.2 years (29–72 years). All women were parous with a mean gravid and parity of 5.5 ± 2.66 and 3.4 ± 2.06, respectively. All women were given birth vaginally except one patient's last birth was delivered with cesarean section. The mean body-mass index of the patients was 24.25 ± 2.43. Nine (45%) patients were in the premenopausal and 11 (55%) patients were in the postmenopausal status.

In all patients' uterine descensus of grades 2–4 was present with additional cystorectocele in 9 patients (45%), cystocele in 6 patients (30%), rectocele in 4 patients (20%), and elangatio colli in 6 patients (30%). In 5 of the patients with elangatio colli concomitant cystorectocele was present. Elangatio colli was treated with Manchester procedure and cystocele and/or rectocele were treated with anterior and/or posterior vaginal wall repair. In two patients (10%) there was stress urinary incontinence which was treated with Burch procedure. The mean operation time for KEPL and vaginal procedures was 39.5 ± 7.93 minutes (30–60 minutes). No intraoperative complications were detected except in one patient light bleeding occurred due to epigastric artery injury. Mean length of hospital stay was 1.64 ± 0.94 days (1–3 days).

Postoperative complications did not develop. No sign of dehiscence or incision infection was detected and none of the patients need transfusion. Analgesia was provided in all patients with nonsteroidal anti-inflammatory agents. All patients' foley catheters were withdrawn on the first postoperative day. Intermittent catheterization was performed in two patients with difficulty in urination. These two patients had spontaneous urination on the 3rd postoperative day with less than 100 cc residual urine. All patients were discharged with oral antibiotics, antiseptic solutions, and non-steroidal anti inflammatory agents. Additionally, local estrogen creams were given for two weeks to postmenopausal women. All patients were advised not to be constipated and to avoid situations that would increase the intra-abdominal pressure at least for three months. Patients were seen on the postoperative 6th week, 3rd, 6th, and 12th months. After the first year, a yearly follow-up was scheduled. Mean follow-up time was 48.95 ± 42.8 months (6–171 months). In all cases uterine descensus was detected to be at the level of ≤ grade 1. In none of these cases any sign of POP or urinary incontinence was detected.

## 4. Discussion

Pelvic floor disorders are emerging with increasing frequency with the prolongation of human life cycle and with medical, social, and economical dimensions it generates serious problems [3, 4, 6, 8]. Multifactorial determinants like race, gender, age, birth, trauma, obesity, increased intra-abdominal pressure, and neuromuscular disorders were involved in the development of fascial deficit as well as weakening of suspensory elements. Uterine prolapse was evolved from the deficit of the uterosacral-cardinal complex [9, 10].

In the US, 600.000 women had hysterectomy for benign causes every year and 40% of women over age 60 were hysterectomized. In recent years, there were increasing evidence and publications about the issue that urinary incontinence and POP frequency were increased after hysterectomy [7, 8]. POP is the third common cause of hysterectomy in the US and every year more than 300.00 operations were performed. In about 30% of these cases, reoperation need arises in later time [4, 7]. From this point of view, hysterectomy for uterine prolapse could be followed with recurrence of POP which emerges as a major issue. In the treatment of POP recurrence, usually meshes were used to lower the recurrence but mesh complications were another important issue of concern [11]. Additionally, urinary incontinence was increased after hysterectomy because of the damage caused to the pelvic nerves and pelvic supportive tissues [7–9]. The absence of uterus which is the key of the vaginal apex further disturbs the apical support and causes urinary incontinence and POP. Particularly, in hysterectomized parous women over age 50, up to 40% of anterior vaginal wall prolapse was monitored over time in proportion [7, 8].

Due to above mentioned reasons, performing hysterectomy just for only POP is controversial today. The pathology of POP is not directly related to the uterus. The main problem is the weakness of supportive and suspensory elements of the pelvic floor and endopelvic fascia [9, 10]. The round ligament is the only stable ligament in uterine prolapse that it was used for many years in suspension operations via transperitoneal, laparotomy, or laparoscopic approaches [9, 10]. However, most of these operations had high recurrence rates. In laparoscopic suture hysteropexy and abdominal sacrohysteropexy series recurrence prolapse rate is reported to be 19–21% and 5–6.6%, respectively [12–14]. While the recurrence rate appeared to be low in the abdominal route, severe gastrointestinal complications could occur due to mesh erosion in this approach. Laparoscopic extraperitoneal uterine suspension to the anterior abdominal wall had a recurrence rate of 6% at the end of one year [15]. Reoperation incidences in laparoscopic uterosacral hysteropexy series and in laparoscopic hysterectomy with uterosacral colpopexy series were 28% and 21%, respectively [16].

In our series, concomitant defect to uterine prolapse was restored at optimal level vaginally. In young and sexually active women, while preserving the integrity of the perineal skin, levator plication was avoided. In the present literature, levator plication was reported to cause dysparennia in 12–27% of cases [17, 18]. Suprapubic extraperitoneal round ligament suspension was performed without opening the parietal peritoneum. Intact peritoneum and no mesh use provided postoperative fast and uncomplicated recovery. Mesh used intraperitoneal suspension procedures were with high morbidity (e.g., ileus, uretery complications and intra-abdominal adhesions) rates [19–21].

Advantages of KEPL include short mean operation time, regional anesthesia option, extraperitoneal approach to avoid from all intraperitoneal complications, and implementation of simultaneously urinary incontinence surgery like Burch procedure. Additionally, KEPL is open for laparoscopic development and implementation.

Reoperation for recurrence after POP surgery is a major issue for important part of the patients [22]. The most important success of KEPL was that no recurrence occurred during an average of 4 years of followup. Today, with increased life expectancy, quality of life comes to the fore, so in addition to the procedure of restoring the existing anatomical defect, both for physiological and psychological aspects, it is important to protect the uterus.

## 5. Conclusions

In POP, when appropriate patient selection criteria are met, KEPL ligamentopexy using the healthy round ligament for suspension of the uterus in addition to the repair of the damaged pelvic floor and/or uterosacral and cardinal ligaments has a low morbidity and high efficiency in POP surgery and it is a candidate to become an alternative treatment to abdominal, vaginal, or laparoscopic sacrocolpopexy operations. Additionally, prospective randomized controlled trials are needed to compare it with other methods.
